# Computational model of midbrain dopaminergic neuron activity in ageing and obesity

**DOI:** 10.1186/1471-2202-14-S1-P85

**Published:** 2013-07-08

**Authors:** Svitlana Popovych, Ursel Collienne, Simon Hess, Peter Kloppenburg, Silvia Daun-Gruhn

**Affiliations:** 1 Emmy-Noether Research Group of Computational Biology, Department of Animal Physiology, University of Cologne, 50674 Cologne, Germany; 2 Cologne Excellence Cluster on Cellular Stress Responses in Aging-Associated Diseases, Department of Animal Physiology, University of Cologne, 50674 Cologne, Germany

## 

Obesity is an increasing health problem in the modern world. Feeding behavior is mostly controlled by homeostatic and hedonic systems. It was already demonstrated in the hypothalamus that diet-induced obesity can change the spontaneous activity of cells involved in homeostatic regulation. It is, however, unclear if the hedonic regulation is also affected by diet-induced obersity. The midbrain dopaminergic (DA) neurons are a key component of the hedonic system. Usually, dopaminergic neurons in brain slice preparations show a highly regular pacemaker-like activity pattern. However it was found in [1], that in mice fed on high fat diet (HFD) a significantly increased proportion of DA neurons fired irregularly as compared to the ones in mice that ware fed on normal diet (NCD). A mathematical model of midbrain dopaminergic neuron (DA) has been developed to better understand the mechanisms underlying the different types of firing patterns that these cells exhibit in vitro. The dopaminergic neuron was modeled using a single compartment which includes voltage and Ca^2+^-dependent currents described by Hodgkin-Huxley kinetics. The model used in this study is based on an existing DA neuron model [2,3] and some parameters were determined using new voltage-clamp data from HFD and NCD mouse brain slices.
